# Contextual control over expression of fear is affected by cortisol

**DOI:** 10.3389/fnbeh.2012.00067

**Published:** 2012-10-11

**Authors:** Vanessa A. van Ast, Bram Vervliet, Merel Kindt

**Affiliations:** ^1^Department of Clinical Psychology, University of AmsterdamAmsterdam, Netherlands; ^2^Psychology of Learning and Experimental Psychopathology, Department of Psychology, University of LeuvenLeuven, Belgium

**Keywords:** cortisol, fear-conditioning, fear potentiated startle, context, anxiety disorders, sex differences

## Abstract

At the core of anxiety disorders is the inability to use contextual information to modulate behavioral responses to potentially threatening events. Models of the pathogenesis of anxiety disorders incorporate stress and concomitant stress hormones as important vulnerability factors, while others emphasize sex as an important factor. However, translational basic research has not yet investigated the effects of stress hormones and sex on the ability to use contextual information to modulate responses to threat. Therefore, the purpose of the present study was threefold: first, we aimed at developing an experimental paradigm specifically capable of capturing contextual modulation of the expression of fear. Second, we tested whether cortisol would alter the contextualization of fear expression. Third, we aimed at assessing whether alterations in contextualization due to cortisol were different for men and women. Healthy participants (*n* = 42) received placebo or hydrocortisone (20 mg) prior to undergoing a newly developed differential contextual fear-conditioning paradigm. The results indicated that people rapidly acquire differential contextual modulation of the expression of fear, as measured by fear potentiated startle (FPS) and skin conductance responses (SCR). In addition, cortisol impaired the contextualization of fear expression leading to increased fear generalization on FPS data in women. The opposite pattern was found in men. Finally, as assessed by SCR, cortisol impaired differential conditioning in men. The results are in line with models suggesting heightened vulnerability in women for developing anxiety disorders after stressful events.

## Introduction

The predominant experimental model for the pathogenesis of human anxiety disorders is that these disorders originate from a learned association between a previously neutral event conditioned stimulus (CS), and an anticipated disaster unconditioned stimulus (US) (Mineka and Oehlberg, 2008). Accordingly, in a typical Pavlovian conditioning procedure a discrete stimulus such as a tone or a light is contingently presented with an US (e.g., an electrical shock) (e.g., Kindt et al., 2009). However, from a real-life perspective, environmental challenges constantly change and thus demand for flexible and adaptive expression of fear learning and expression (Schiller et al., 2008). When this flexible adaptation of fear expression is disrupted, pathological conditions may develop. For instance, patients with posttraumatic stress disorder (PTSD) respond to trauma-related danger cues even in objectively safe environments, apparently unable to adequately modulate their responses based on the contextual cues present. Indeed, it is often emphasized that at the core of anxiety disorders is the inability to use contextual information (i.e., safety signals) to modulate behavioral responses to a potentially threatening event (Ehlers and Clark, 2000). More specifically, it has been suggested that dysregulation in the *contextualization* of fearful memories may be an important vulnerability factor for developing PTSD (Liberzon and Sripada, 2008; Cohen et al., 2009; Acheson et al., 2011). Thus, research into underlying mechanisms of anxiety disorders should incorporate contexts as an important modulator of simple CS-US associations.

Situations in which discrete cues are predictive of threat only under certain conditions are referred to as occasion setting (Schmajuk and ABuhusi, 1997). A procedure employing contexts as occasion setter could be a promising model to test differential contextual control over the expression of fear. Though several studies have investigated the context dependency of extinction (e.g., Milad et al., 2005; Vansteenwegen et al., 2005; Effting and Kindt, 2007; Neumann and Kitlertsirivatana, 2010), far less studies have touched upon the topic of occasion setting (Baeyens et al., 2001, 2004; De Houwer et al., 2005). Even more important, none of those studies have incorporated physiological -dependent variables like fear potentiated startle (FPS) even though these form the predominant model of conditioned fear responses (Hamm and Weike, 2005; Mineka and Oehlberg, 2008). Therefore, the first aim of the present study was to adapt a typical occasion setting paradigm in such a way that assessment of contextual modulation of FPS data would be possible.

Several models for the etiology of anxiety disorders incorporate stress and associated stress hormones such as cortisol as important vulnerability factors (Korte, 2001; Elzinga and Bremner, 2002; Shin and Liberzon, 2009). Corticosteroids are central modulators of human cognition, like learning and memory (Lupien et al., 2002; Het et al., 2005; Wolf, 2008) and exert their effects by binding to high affinity mineralocorticoid receptors (MR) and to lower affinity glucocorticoid receptors (GR) (Joëls et al., 2008). Though ubiquitously present in the (animal) brain, both MR and GR receptors are especially densely situated in the amygdala and hippocampus (Joëls and Baram, 2009), with GR concentrations situated in the medial prefrontal cortex as well (Herman et al., 2005). These three regions play a fundamental role in the regulation of conditioned fear expression in both animals (Barbas et al., 2003) and humans (Hartley and Phelps, 2009; Shin and Liberzon, 2009) and alterations in this network, including the hippocampus, have repeatedly been related to corticosteroid actions as well as anxiety disorders (Shin and Liberzon, 2009). Indeed, both animal and human studies illustrate that stress and/or corticosteroids can alter simple associative fear learning (Rodrigues et al., 2009; Wolf et al., 2012), but the effects of corticosteroids on the inability to restrict fear responses to the appropriate predictors has been largely neglected in research. Only very recently one study has shown in mice that induction of glucocorticoids into the hippocampus after fear-conditioning decreased the ability to restrict fear to the appropriate context (Kaouane et al., 2012). In humans one study has shown impairment in conditional discrimination learning after social stress exposure, which was associated with endogenous cortisol (Wolf et al., 2012), but it is yet unknown how corticosteroids may alter the contextual control over expression of fear. Therefore, a second aim of the present study was to assess whether cortisol may cause dysregulation of contextual control in the expression of fear.

Numerous animal studies have shown alterations by stress on hippocampal-dependent tasks such as context conditioning or trace-conditioning (Cordero et al., 2003; Bangasser and Shors, 2004; Weiss et al., 2005). However, some of these studies found impairing effects, while others have found enhancing effects. One likely explanation for these contradictions may lie in sex differences in sensitivity to stress and associated corticosteroids. In animals, fear-conditioning and other associative learning processes diverge strongly between the two sexes (Dalla and Shors, 2009). When stress comes into the picture, females showed reduced conditioned responses on a hippocampal-dependent trace-conditioning procedure (Bangasser and Shors, 2004), while males showed enhancements (Weiss et al., 2005). In humans, cortisol administration (Kuehl et al., 2010) or endogenous secreted cortisol by social stress (Duncko et al., 2007) enhanced performance on a trace eye-blink-conditioning task in men. But another study found impairment by cortisol administration in both men and women (Nees et al., 2008), or even no effect at all (Vythilingam et al., 2006). These contradictions notwithstanding, it is important to realize that within the context of etiological models of anxiety disorders sex specific sensitivity to stressful events has been repeatedly associated with the higher prevalence of mood and anxiety disorders in women (Kessler et al., 1993; Trentani et al., 2003; Cahill, 2006). Therefore, a final aim of the present experiment was to assess sex effects in the contextual modulation of fear expression by cortisol.

**Figure 1:**
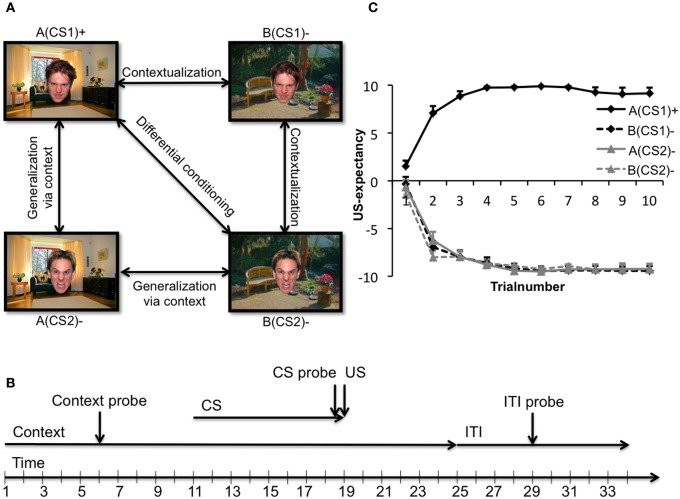
**(A)** Experimental conditioned stimuli and relevant comparisons. An image of a living room and an image of a garden constituted the safe (context B) and threat (context A) contexts, while two angry faces constituted the CS+ (CS1) and CS− (CS2). Several comparisons of interest concerning fear contextualization and fear generalization exist. A(CS1)+ > B(CS2)− denotes differential conditioning; A(CS1)+ > B(CS1)− denotes proper fear contextualization; B(CS1) > B(CS2) denotes impaired contextualization; A(CS1)+ > A(CS2)− denotes less fear generalization via the context; A(CS2)− > B(CS2)− denotes fear generalization via the context. **(B)** Timeline of an exemplary trial. Each trial started with the onset of a context (duration: 25 s), in which after 11 ± 1 s a face appeared (duration: 8 s) and again disappeared. After face offset, the context remained onscreen for another 6 s (± 1), followed by a variable inter trial interval (ITI: 9 ± 1 s). If a context startle probe was presented, it occurred after 6 ± 1 s relative to context onset. During every CS presentation a CS probe was presented after 7.5 s. ITI probes were presented in the middle of the ITI trial. **(C)** US-expectancies. Mean expectancy scores of the unconditioned stimulus as a function of stimulus type across all groups. Error bars represent standard errors of the mean. All participants were completely aware of the experimental contingencies.

A novel experimental paradigm, inspired on the principles of an occasion setting paradigm, was designed in order to directly assess the ability of participants to discriminate between safe and dangerous environments. Shortly, two angry faces served as CSs (CS1 and CS2) that were alternately presented in two different contexts (background pictures of a living room and garden; context A and context B). Only the combination of context A (the “threat” context) with CS1 [together denoted as: A(CS1)+] was followed by the US (i.e., an electric shock). The same CS presented in context B [the “safe” context, together denoted as: B(CS1)−] was not followed by a US. Furthermore, as control, the second CS (CS2) was presented in both the threat and safe contexts as well [denoted as A(CS2)− and B(CS2)−, respectively]. For examples of these stimuli, see Figure 1A. Expression of fear was measured with FPS and skin conductance response (SCR). However, these dependent variables reflect rather distinctive aspects of conditioned responses: SCR is associated with contingency learning, whereas the FPS supposedly is a rather specific measure of fear (Hamm and Weike, 2005; Mineka and Oehlberg, 2008; Soeter and Kindt, 2010). In addition, because FPS can be shown in both animals and humans and underlying neurocircuitry has been highly preserved cross-species (Grillon, 2008), it poses an excellent tool for translational research.

Based on animal data showing impairment of corticosteroids in females but enhancement in males on a trace-conditioning task (Bangasser and Shors, 2004; Weiss et al., 2005), we hypothesized that cortisol would impair the contextualization of fear expression in women, but enhance it in men. In the current paradigm, correct contextualization of fear expression boils down to enhanced fear responding to A(CS1)+, with the other three stimulus combinations eliciting equally less fear. However, with reduced fear contextualization, fear will generalize to the CS1 in the safe context. In addition, fear from the threat context itself may generalize to the safe CS2 in the threat context. This is to be expected because contexts may acquire a direct association with the US (Rescorla and Wagner, 1972) as well, which indeed has been shown in humans (Baas et al., 2004). Thus, associative strength of B(CS1)− and A(CS2)− with the US will increase. Taken together, a downward generalization gradient in fear responding is expected as stimulus divergence increases from A(CS1)+ through B(CS1)−, through A(CS2)−, with B(CS2) expected to elicit least fear. With impaired contextualization of fear, and thus enhanced generalization, the gradient from A(CS1)+ through B(CS2)− will approach linearity (Lissek et al., 2008, 2010).

## Methods

### Participants

In total 46 participants gave written informed consent and subsequently completed the study. Informed consents were archived by the first author. The study was approved by the local ethical committee of the University of Amsterdam and performed in accordance with ethical standards laid down in the Declaration of Helsinki. Inclusion criteria as assessed by self-report were no past or present psychiatric or neurological condition and age between 18 and 35 years. In addition, participants having any somatic or endocrine disease (e.g., acute asthma), or taking any medication known to influence central nervous system or endocrine systems were excluded from participation. Participants were asked to refrain from smoking, caffeine intake, eating, and 2 h heavy exercise before participation. In order to reduce variance in the female group, all women included in the study were taking birth-control (only monophasic preparations), and were tested only during “on phase” of pill intake, which is general practice in this field (c.f., Stark et al., 2006; Tabbert et al., 2010; Merz et al., in press). Participants were rewarded for their participation with course credits or were paid a small amount (€ 21) of money.

After conditioning, two participants could not verbally report the experimental contingencies (confirmed by their online US-expectancy ratings and post-experimental questionnaire), and one participant reported having used drugs prior to participation. These three participants were excluded from any further analyses. The final sample consisted of 43 participants divided into four subgroups: cortisol women (*n* = 11), placebo women (*n* = 11), cortisol men (*n* = 10), and placebo men (*n* = 11). Mean overall age was 21.3 years (*SD* = 3).

### Physiological and psychological measures

#### Drug administration and assessment

In between-subjects, double-blind study design, participants were randomly assigned to either the hydrocortisone or placebo group. Hydrocortisone and placebo (albochin) were manufactured into identical appearing capsules by a local pharmacy. A single dose of 20 mg of hydrocortisone was employed to elevate endogenous cortisol to a level equivalent to moderate acute stress (Abercrombie et al., 2003).

To assess salivary free cortisol concentrations of each subject, participants were asked to lightly chew on Salivette collection devices (Sarstedt, Nümbrecht, Germany) for about a minute, until it was completely saturated. Saliva samples were collected immediately before drug administration, as well as before and after the fear-conditioning procedure. After testing, the salivettes were stored at −30°C. Upon completion of the entire study, samples were sent out to Dresden (Prof. Dr. Kirschbaum, Technische Universität, Dresden, Germany) for biochemical analysis. There, salivary free cortisol concentrations were measured using a commercially available chemiluminescenceimmuno-assay (CLIA) with high sensitivity of 0.16 ng/ml (IBL, Hamburg, Germany).

#### US-expectancy ratings

Participants were asked to continuously rate their US-expectation throughout the entire conditioning phase, thus enabling us to collect ratings originating from both the context and CS presentations. Ratings were given by sliding a lever on a box (custom made from a joystick) that on its turn operated a cursor on a scale that showed at the bottom of the computer screen. The scale was continuous and depicted 11 points labeled from “certainly no electrical stimulus” (−5) through “uncertain” (0) to “certainly an electrical stimulus” (5). Expectancy data were sampled at 1000 S/s. US-expectancy ratings were recorded with the software program VSSRP98 v6.0 (Versatile Stimulus Response Registration Program, 1998; Technical Support Group of the Department of Psychology, University of Amsterdam).

#### Fear potentiated startle

Startle stimuli to probe the FPS reflex were 104 dB, 40 ms bursts of white noise with a near instant rise time and delivered binaurally through headphones (Sennheiser, model HD 25-1 II). Sound pressure and dB level was calibrated using a sound level meter (Rion, NA-27, Japan). Conditioned eye-blink reflexes probed by the loud acoustic stimulus were measured through electromyography (EMG) of the left orbicularis oculi muscle. Hereto the skin under the eye and on the forehead was disinfected with some alcohol to reduce resistance of the skin. Two 6 mm Ag/AgCl electrodes filled with a conductive gel (Signa, Parker) were placed approximately 1 cm under the pupil and 1 cm below the lateral canthus, as set by the standards of Fridlund and Cacioppo (1986). A ground electrode was placed on the forehead, 1 cm below the hairline (Blumenthal et al., 2005). The EMG amplifier was designed and built by the technical support group of the UvA Psychology department and consisted of two stages. The pre-amplifier had an input resistance of 10,000 Ω. The EMG signal was set at a frequency response of DC-1500 Hz and was then amplified by 200. A 50 Hz notch filter was used to reduce interference from the mains noise. Then the signal was amplified with a variable amplification factor of 0–100 times. Finally, the EMG signal was digitized at a rate of 1000 S/s. Startle responses were recorded with the software program VSSRP98 v6.0 (Versatile Stimulus Response Registration Program, 1998; Technical Support Group of the Department of Psychology, University of Amsterdam).

#### Skin conductance response

Electrodermal activity was measured by two curved Ag/AgCl electrodes of 20 by 16 mm that were attached with adhesive tape to the medial phalanges of the first and third fingers of the left hand. The in-house built amplifier applied a sine-shaped excitation voltage (1 V peak–peak) of 50 Hz derived from the mains frequency to the electrodes in order to detect changes in the electrodermal activity. The signal from the input device was led through a signal-conditioning amplifier. The analogue output was digitized at 1000 S/s by a 16-bit AD-converter (National Instruments, NI-6224). SCR were recorded with the software program VSSRP98 v6.0 (Versatile Stimulus Response Registration Program, 1998; Technical Support Group of the Department of Psychology, University of Amsterdam).

### Subjective measures

Participants filled out Dutch translations of the trait portion of the state-trait anxiety inventory (STAI-T; Spielberger et al., 1970; Van Der Ploeg et al., 1980), perceived stress scale (PSS; Cohen et al., 1983; De Vries, 1998) and the survey of recent life events (SRLE; Kohn and Macdonald, 1992; Majella De Jong et al., 1996). Furthermore, to assess the influence of hydrocortisone on self-reported affective state, participants filled out the state-anxiety inventory (STAI-S; Spielberger et al., 1970; Van Der Ploeg et al., 1980) and the positive affect and negative affect schedule (PANAS; Watson et al., 1988). Subjective evaluation of the conditioned stimuli on arousal and valence dimensions was assessed online using self-assessment manikins (SAM; Bradley and Lang, 1994). Finally, a post-experimental questionnaire was given in which participants rated startle probe intensity and US-intensity both assessed on an 11-point scale ranging from −5 (unpleasant) to 5 (pleasant). Also, participants had to indicate which context-face combination was followed by the US. Finally, participants indicated which substance they thought they had received.

### Experimental task

Two clipped-out pictures of male angry faces (Tottenham et al., 2009) served as conditioned stimuli. These two stimuli were matched on validity and reliability of their facial expression. The two CSs were 105 mm wide and 136 mm high, and alternately presented against one out of two possible background images. These background images were a picture of a living room and a garden that filled the screen of the 19-in. computer monitor entirely. Assignment of the faces as CS1 or CS2 and assignment of the background pictures as context A or B were counterbalanced across all participants. Only the unique combination of the “threat” context A with CS1 [denoted A(CS1)+] was followed by the US (at a 100% reinforcement rate), while A(CS2)− was not, neither were the two CSs in combination with the “safe” context B [B(CS1)− and B(CS2)−] ever followed by the US. Prior to fear-conditioning, participants were presented with eight startle probes (“noise alone,” NA) with an inter-probe interval of 15–19 s, to allow blink responses to habituate. During fear-conditioning, each one out of the four possible CS-context combinations was presented 10 times, amounting to a total of 40 trials. In addition, 10 ITI startle probes, 20 context A probes and 20 context B probes were presented. The four different trial-types, CS probes, context probes, and ITI probes were randomly shuffled within blocks of four trials. Thus, every four trials one A(CS1)+, one A(CS2)−, one B(CS1)−, and one B(CS2)− trial was presented (all CSs being consistently presented along with a probe), as well as one ITI probe, two context A probes and two context B probes. Contexts were always presented for a total duration of 25 s. If a context probe was presented, it was always presented 6 ± 1 s after context onset. The facial CS appeared after 11 ± 1 s relative to context onset. CSs were always presented for 8 s. Relative to CS onset a startle probe was presented after 7.5 s, followed by the US after 0.5 s (CS and US co-terminated). ITIs (time in between contexts) took 9 ± 1 s on average. See Figure 1B for a schematic trial outline. The US consisted of transcutaneous electrical stimulation for 2 ms to the upper side of the left wrist. USs were given through a pair of custom made Ag electrodes of 20 by 25 mm with a fixed inter-electrode mid-distance of 45 mm, controlled by a Digitimer constant current stimulator model DS7A (Hertfordshire, UK). Electrolyte gel (Signa, Parker, USA) was applied between the skin and the electrodes.

### Procedure

Participants were instructed to refrain from smoking, eating, or drinking (except for water) 2 h prior to participation. To control for diurnal variation in general cortisol levels, all testing took place between 12.00 am and 7.00 pm when cortisol levels are typically low (Pruessner et al., 1997). All testing took place in a sound-attenuated room, situated adjacent to the experimenter room. Upon arrival, participants read the information brochure, were medically screened, signed the informed consent and filled out the STAI-T, PSS, and SRLE questionnaires. Then, the ECG electrodes were attached and a 10-min baseline ECG recording (data not further analyzed) took place, during which participants watched a fragment of a relaxing movie (“Coral see dreaming,” Hannan, 1999). Next, baseline mood questionnaires (PANAS and STAI-S) along with a baseline saliva sample were taken. For saliva sampling participants were instructed to lightly chew on the salivette for at least a minute until it was completely saturated with saliva. Then, participants received either 20 mg hydrocortisone or placebo pill, in a double-blind way. In order to reach peak plasma levels about halfway of the conditioning phase (Czock et al., 2005), a resting period of 45 min was then implemented. While waiting, participants read magazines. After EMG, SCR, and shock electrode attachment, a shock workup procedure was completed to establish a level of shock that was “unpleasant, but not painful.” Participants were explicitly told to learn to predict whether they would receive an electrical stimulus or not based on the specific combinations of faces and background images. Awareness is known to be an important modulator of fear expression (Grillon, 2002; Jovanovic et al., 2006). By giving explicit instructions prior to conditioning, we aimed at reducing variability in our sample due to differences in contingency learning across the sample. Right before beginning of the fear-conditioning phase, participants again filled out the PANAS and STAI-S questionnaires, along with a second saliva sample. Conditioning took approximately 20 min, after which participants completed the SAM ratings online, filled out the PANAS and STAI-S along with a final saliva sample, and completed the experiment by filling out the post-experimental questionnaire.

### Data reduction and analysis

In order to assess group differences in sample characteristics univariate ANOVAs were employed with the between subject factors Sex (men and women) and Drug (hydrocortisone, placebo). To assess the effect of hydrocortisone administration and sex on salivary cortisol levels, a mixed ANOVA with the within-subject factor Time (baseline, pre-conditioning, and post-conditioning) and between-subject factors Sex and Drug was conducted.

To maximize signal-to-noise ratio, raw EMG data were conditioned to a band-pass between 28 Hz and 500 Hz (Butterworth, 4th order; Blumenthal et al., 2005) and subsequently rectified. The onset latency window for the blink reflex was 0–120 ms and the peak magnitude was determined within 20–150 ms following probe onset. Outliers were defined (*Z* > 3) and replaced by linear trend at point. SCR to the CSs and contexts were obtained by subtracting the baseline (1 s average before stimulus onset) from the maximum absolute SCR score obtained from a window of 1–7 s following stimulus onset. Thus, all SCRs to the facial CSs reflect changes in SCR over and above any changes produced by the context. Raw SCR scores were range corrected (Lykken, 1972) and subsequently corrected to be normally distributed (Log 2 + SCR). Only participants showing SCR responses were included in the analysis (with SCRs ≥ 0.03 μS in 2 or more of the 40 trials (Milad et al., 2005). Outliers were defined (*Z* > 3) and replaced by linear trend at point. To obtain US-expectancy ratings for the CSs and the contexts concomitant to the startle responses, 1 s averages were calculated right before each startle probe onset.

In order to reduce variability of EMG, SCR, and expectancy responses, data were averaged into blocks of two trials. Startle response to NA trials were analyzed with use of mixed repeated measures ANOVAs with Drug and Sex as between-subject factor and block (block 1 through 5) as within-subject factors. FPS, SCR, and US-expectancy data were then first subjected to mixed ANOVAs, again with Drug and Sex as between-subject factors and Block number and CS type [A(CS1)+, B(CS1)−, A(CS2)−, B(CS2)−] as within-subject factors. Following up on significant interaction(s) with Drug, differences in contextualization gradients in data for the four groups were assessed. Hereto, averages of the final half of acquisition (i.e., consisting of five trials) for the four different CSs were calculated. We expected these averages to reflect changes in conditioned responding due to learning over the earlier fear-conditioning trials, as has been reported previously (Labar et al., 2004). Then, we ran an omnibus mixed ANOVA with the within-subject factor CS type and between subjects-factor Sex and Drug to assess the amount of within-group fear contextualization from A(CS1)+ through B(CS2)− (Lissek et al., 2008, 2010). Following up on that analysis, quadratic trend analyzes were run in all groups separately, with the a priori hypothesis that cortisol in women would cause a departure from the quadratic function expected in their controls, while in men cortisol would cause an enhancement in the gradient. Finally, to follow-up on the altered contextualization gradients and thereby reveal specific differences in contextualization, several planned comparisons within each subgroup of participants were run, as well as comparisons between groups. More specifically, the effects of cortisol on general differential conditioning [A(CS1)+ vs. B(CS2)−], fear contextualization [A(CS1)+ vs. B(CS1)− and B(CS1)− vs. B(CS2)−], and fear generalization via the context [(A(CS2)− vs. B(CS2)− and A(CS1)+ vs. A(CS2)−] were assessed (see Figure 1A for all the relevant comparisons). A Greenhouse-Geisser procedure was used in case of violation of the sphericity assumption in ANOVAs. The alpha level was set at 0.05 for all statistical analyses.

## Results

### Participant characteristics

There were no main effects of Sex or Drug nor any interaction between the two in terms of BMI, age, reported trait anxiety, PSS, the SRLE, shock intensity or experienced intensity of the shock (all *F*s < 2.1, *p* = *ns*.).

### Salivary cortisol

In line with expectations, administration of hydrocortisone significantly increased salivary cortisol levels comparable to real-life severe psychological stress (Abercrombie et al., 2003), as evidenced by a significant interaction of Time × Drug [*F*_(2, 78)_ = 17.85, *p* = 0.000, η^2^_p_ = 0.314]. In addition, significant main effects of Time and Drug emerged [respectively, *F*_(2, 78)_ = 16.97, *p* = 0.000, η^2^_p_ = 0.303; *F*_(1, 39)_ = 22.82, *p* = 0.000, η^2^_p_ = 0.369]. No interactions with Sex or a main effect of Sex emerged (all *F*s < 0.88, *p* = *ns*.) indicating salivary cortisol levels were at all times comparable for men and women. Specific planned comparisons showed that salivary cortisol levels were elevated in the Drug group both before fear-conditioning (*p* = 0.000) and after fear-conditioning (*p* = 0.000), as compared to the control group. Salivary cortisol levels over the courese of the study are described in Table 1.

**Table 1:** **Salivary cortisol levels over the course of the study (mean ± SE)**.

**Time**	**Placebo (nmol/L)**	**Hydrocortisone (20 mg) (nmol/L)**
Baseline	7.81 ± 0.72	12.05 ± 1.60
Pre-conditioning	6.10 ± 0.83	170.35 ± 38.35
Post-conditioning	5.5832 ± 0.52	158.86 ± 30.43

### US-expectancies

#### US-expectancy during CS presentation

A main effect of CS type indicated that collapsed over all acquisition, expectancies varied for the different CS types [*F*_(3, 117)_ = 869.06, *p* = 0.000, η^2^_p_ = 0.958], indicating successful fear contingency learning for all groups. Contingency learning of the different CS types changed over acquisition showing a typical learning curve, evidenced by an interaction between Block and CS type [*F*_(12, 468)_ = 48.462, *p* = 0.020, η^2^_p_ = 0.554]. None of the other interactions reached significance (all *F*s < 1.23, *p* = *ns*.), neither were there any between subjects-effects significant (all *F*s < 1.93, *p* = *ns*.). Figure 1C depicts the US-expectancy ratings as a function of CS type for all participants.

#### US-expectancy during context presentation

Finally, we tested whether cortisol, sex or a combination of the two did modulate US-expectancies during context presentations. This analysis revealed a main effect of Context [*F*_(1, 39)_ = 31.17, *p* = 0.000, η^2^_p_ = 0.451], indicating that US-expectancies during the threat occasion setting context A was generally higher than during the safety occasion setting context B. None of the other interactions reached significance (all *F*s < 2.0, *p* = *ns*.), neither were there any between subjects-effects significant (all *F*s < 2.25, *p* = *ns*.).

### Fear potentiated startle

#### Contextualization of fear

Analysis of the noise alone trials showed that over complete acquisition, hydrocortisone did not affect startle magnitude [*F*_(1, 39)_ = 0.014, *p* = *ns*.], nor did hydrocortisone influence the course of responding [*F*_(1, 39)_ = 0.236, *p* = *ns*.]. None of the other interactions were significant either (all *F*s < 0.85, *p* = *ns*.). Men showed, however, generally smaller baseline startle responding than women [*F*_(1, 39)_ = 5.187, *p* = 0.028, η^2^_p_ = 0.117]. In order to make male and female responding comparable, we subtracted the NA trials from startle responding to the CSs for all further analyses (Jovanovic et al., 2005).

**Figure 2:**
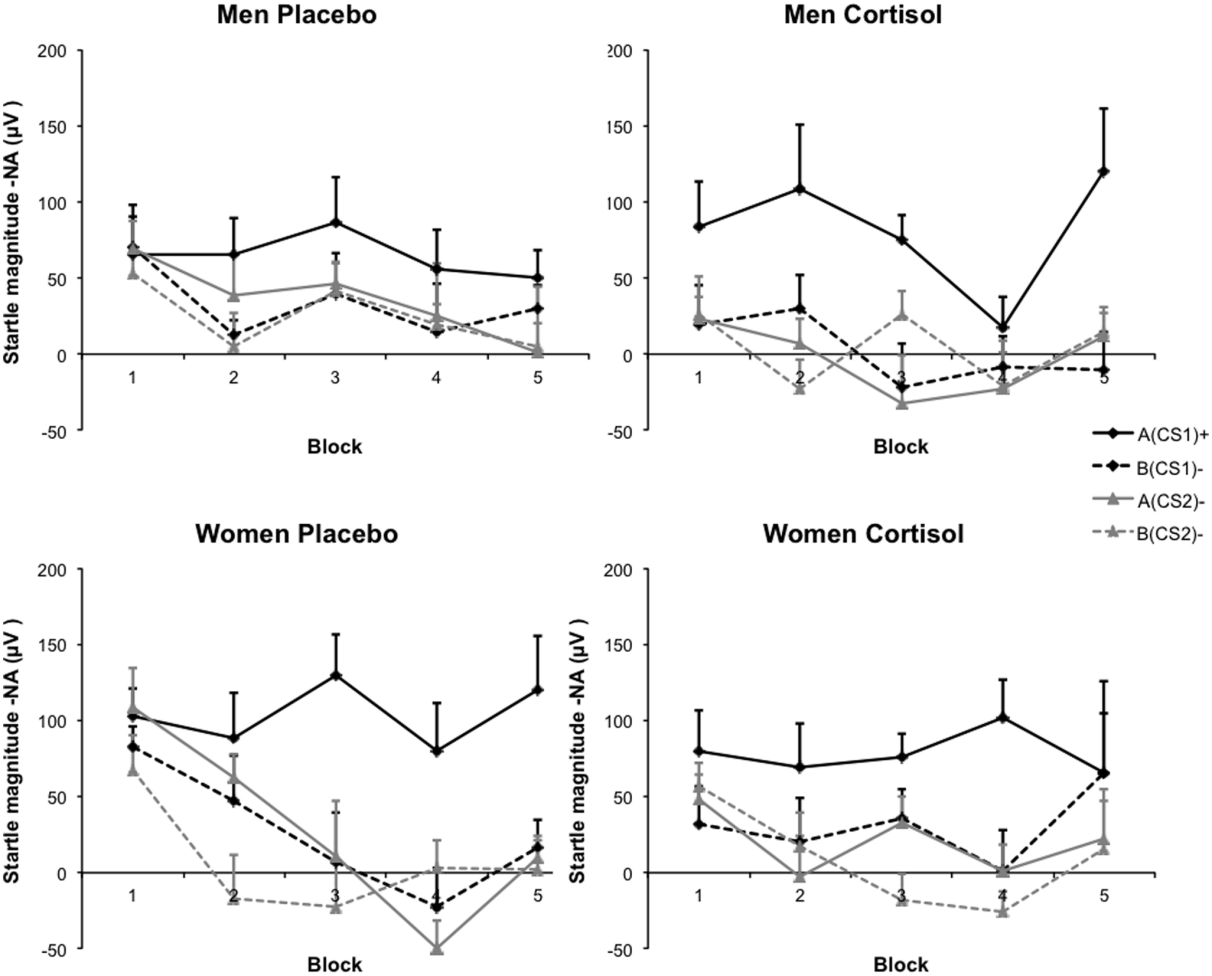
**Startle magnitudes.** Averages of noise alone (NA) trials derived from the same block as the CS presentations were subtracted from average startle potentiation during CS presentations. Blocks consisted of two trials of each CS type. Graphs depict data as a function of Sex and Drug. Error bars represent standard errors of the mean.

Analysis of the relationships among Drug, Sex, CS type, and Block, revealed a main effect of CS type, indicating successful fear acquisition over all groups [*F*_(3, 117)_ = 39.70, *p* = 0.000, η^2^_p_ = 0.504]. Collapsed over all CS types, a main effect of Block indicated that startle responding throughout acquisition slightly decreased [*F*_(4, 156)_ = 3.16, *p* = 0.016, η^2^_p_ = 0.075]. Furthermore, startle responding to the different CS types changed over acquisition showing a typical learning curve, evidenced by an interaction between Block and CS type [*F*_(12, 468)_ = 2.032, *p* = 0.020, η^2^_p_ = 0.050]. Finally, regardless of Drug, a significant three-way interaction between Sex, CS type, and Block showed that men and women differed in startle responding to the four CS types over fear-conditioning [*F*_(12, 468)_ = 1.93, *p* = 0.029, η^2^_p_ = 0.47]. Crucial to the hypothesis at hand, startle responding to the different CS types varied as a function of cortisol and sex, as evidenced by a significant three-way interaction between Drug, Sex, and CS type [*F*_(3, 117)_ = 3.14, *p* = 0.028, η^2^_p_ = 0.074]. In addition, this relationship changed over acquisition blocks, indicated by a significant four-way interaction between Drug, Sex, CS type, and Block [*F*_(12, 468)_ = 2.19, *p* = 0.011, η^2^_p_ = 0.053]. None of the other interactions reached significance (all *F*s < 1.23, *p* = *ns*.), neither were there any between-subject effects significant (all *F*s < 1.93, *p* = *ns*.). Figure 2 displays the group differences in FPS data over the course of conditioning.

**Figure 3:**
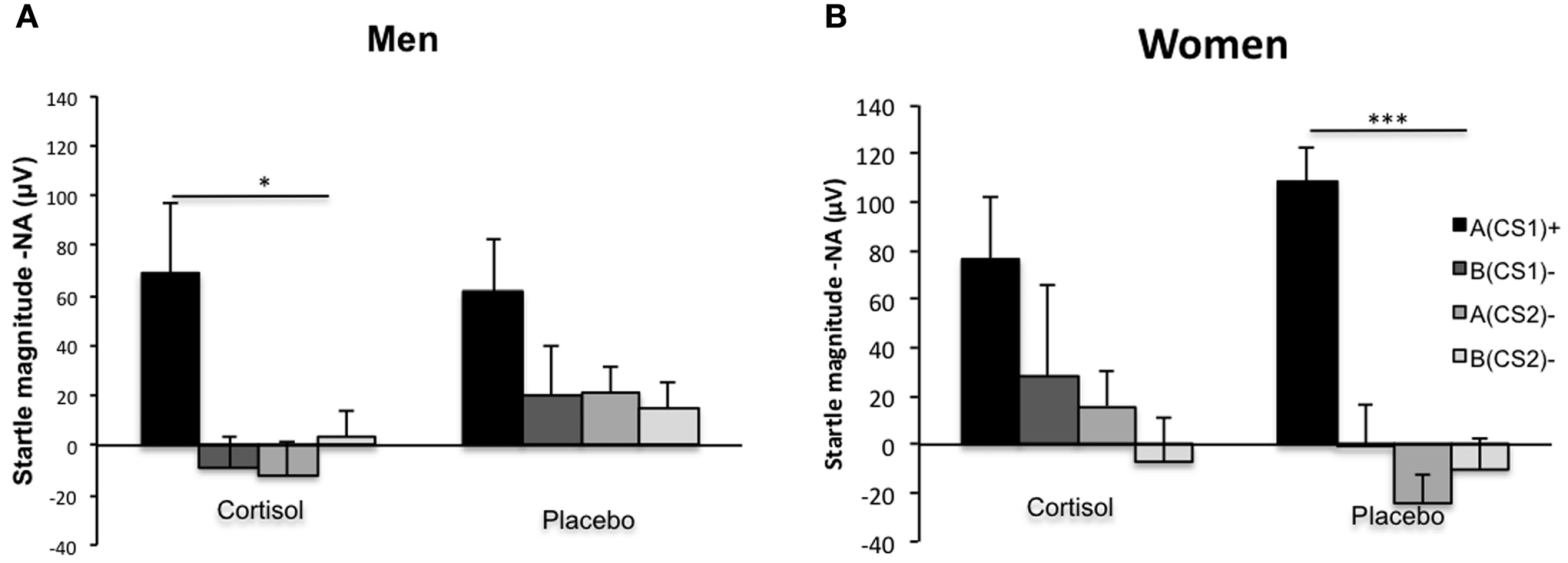
**Fear contextualization and fear generalization.** Deviations from linearity reflect a significant quadratic component in generalization gradients, and are to be expected in the case of proper fear contextualization. (Panel **A**) Shows that men having received the cortisol pill showed proper fear contextualization (significant quadratic component), which was absent after placebo administration, resulting in increased fear generalization (absence of significant quadratic component). (Panel **B**) Shows that women having received the placebo pill showed proper fear contextualization (significant quadratic component), which was absent after cortisol administration, resulting in increased fear generalization (absence of significant quadratic component). Significant quadratic components in generalization gradients are depicted by ^*^p < 0.05; or ^***^p < 0.000.

Following up on the significant interactions with Drug, Sex, CS type, and Block, we aimed at clarifying how exactly cortisol influenced generalization and contextualization in startle responding to the critical CS types in men and women. Hereto, we calculated averages of the final half of acquisition (i.e., consisting of five trials) for the four different CSs. We expected these averages to reflect changes in conditioned responding due to learning over the earlier fear-conditioning trials, as has been reported previously (Labar et al., 2004). In line with the previous analysis the omnibus mixed ANOVA yielded a main effect of CS type [*F*_(3, 117)_ = 32.71, *p* = 0.000, η^2^_p_ = 0.452] indicating a downward generalization gradient in startle responding as stimulus divergence increased from A(CS1)+ to the other CS types. Again, the generalization pattern from A(CS1)+ through B(CS2)− differed across Drug and Sex, evidenced by a significant interaction effect between CS type, Drug, and Sex [*F*_(3, 117)_ = 3.56, *p* = 0.016, η^2^_p_ = 0.084]. Importantly, this effect was attributable to between group differences in the quadratic component of the respective generalization slopes (Interaction between CS type, Drug, and Sex: [*F*_(3, 117)_ = 11.74, *p* = 0.001, η^2^_p_ = 0.231]. Following-up on this interaction, we found that women within the placebo group displayed the expected quadratic generalization gradient [*F*_(1, 10)_ = 40.97, *p* = 0.000, η^2^_p_ = 0.804], while this pattern disappeared after cortisol administration [*F*_(1, 10)_ = 2.59, *p* = *ns*.]. For the men, we observed an opposite pattern: cortisol decreased the amount of generalization, as evidenced by a significant quadratic gradient [*F*_(1, 9)_ = 6.81, *p* = 0.026, η^2^_p_ = 0.431], which was absent in the male placebo group [*F*_(1, 10)_ = 3.44, *p* = *ns*.]. Figure 3 displays the group differences in quadratic gradients.

Though the altered contextualization gradient by cortisol in men and women is informative of the overall pattern of fear responses to the different CS types, it does not significant differences between stimulus types. Therefore, we ran several specific planned comparisons *within* each subgroup of participants. We also compared each relevant difference score *between* the groups. First of all, all subgroups showed significant differential conditioning [A(CS1)+ vs. B(CS2)−; all *p* < 0.039], and cortisol did not alter the amount of differential conditioning in men or in women, as compared to their respective control groups. Further, though all subgroups showed a significant difference between the threat CS1 in the threat context A and the same CS1 in the safe context B [A(CS1)+ vs. B(CS1)−], hydrocortisone significantly reduced this difference in the women (*p* = 0.031), pointing out impaired fear contextualization. In line with this finding, in women cortisol significantly potentiated the threat CS1 within the safe context B as compared to the safe CS2 in the same safe context [B(CS1)+ vs. B(CS2)−; *p* = 0.032], though this effect was not more pronounced than for the control women. Again, within the female cortisol group, fear generalized from the threat context A to the safe CS2 in that same context [A(CS2)+ vs. B(CS2)−; *p* = 0.050]. This was significantly more fear potentiation as compared with the control women (*p* = 0.025). In line with this, the difference between the threat CS1 within the threat context A and the safe CS2 in the same context [A(CS1)+ vs. A(CS2)−] was significantly reduced due to cortisol (*p* = 0.035).

#### Contextual fear

Finally, we aimed at investigating whether cortisol, sex or a combination of the two did modulate startle responding to the occasion setting contexts. This analysis revealed a main effect of Context [*F*_(1, 39)_ = 5.99, *p* = 0.019, η^2^_p_ = 0.133], indicating that startle responding to the threat occasion setting context A was generally higher than to the safety occasion setting context B. None of the other interactions reached significance (all *F*s < 1.23, *p* = *ns*.), neither were there any between subjects-effects significant (all *F*s < 1.93, *p* = *ns*.).

### Skin conductance responding

#### Contextualization of fear

**Figure 4:**
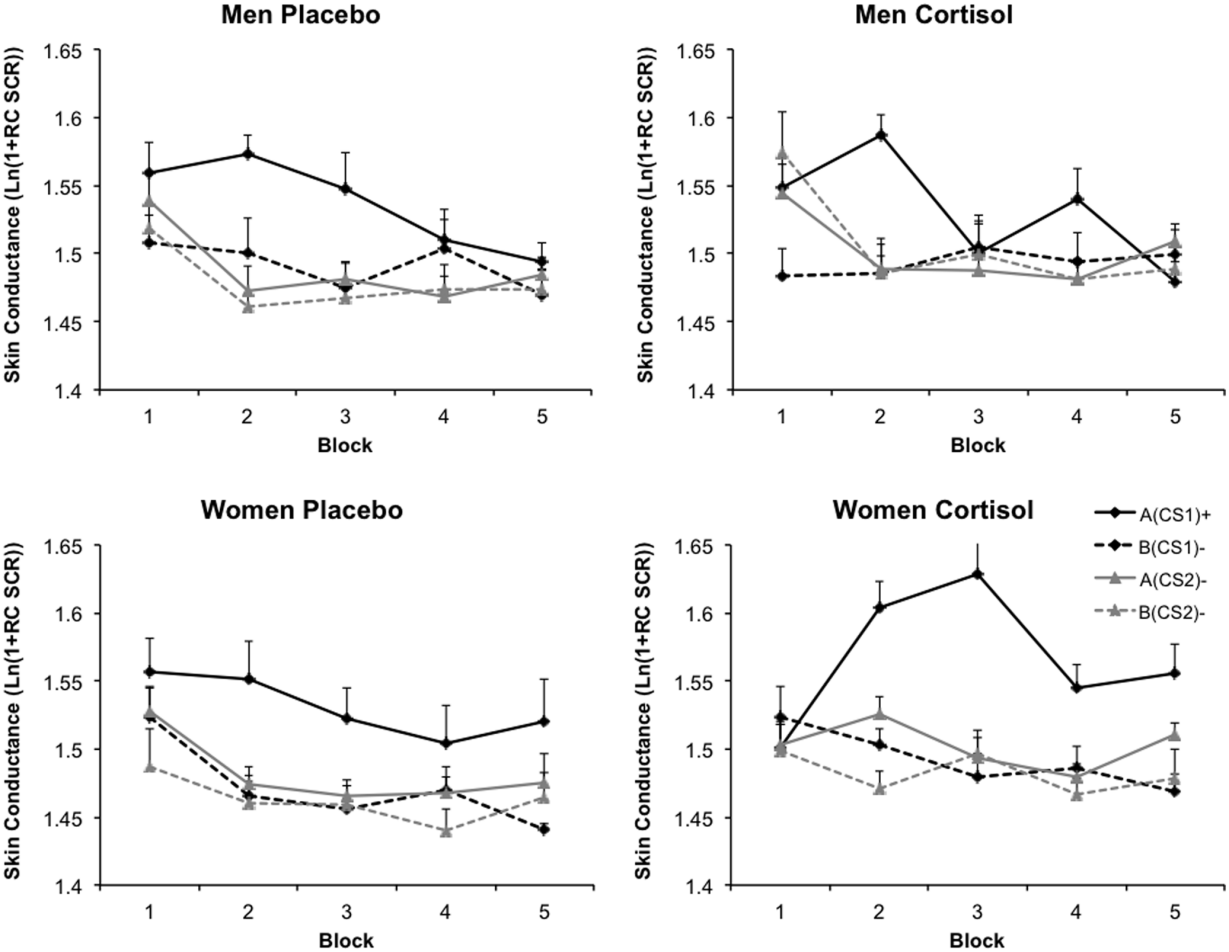
**Skin conductance responses.** Skin conductance data were range corrected and subsequently log transformed. Blocks consist of averages of two trials of each CS type. Graphs depict data as a function of Sex and Drug. Error bars represent standard errors of the mean.

A main effect of CS type indicated that over all acquisition, SCR responses varied for the different CS types [*F*_(3, 108)_ = 48.00, *p* = 0.000, η^2^_p_ = 0.571]. Collapsed over all CS types, a main effect of Block indicated that SCR responding throughout acquisition decreased [*F*_(4, 144)_ = 14.01, *p* = 0.000, η^2^_p_ = 0.280]. Furthermore, SCR responding to the different CS types changed over acquisition indicating successful acquisition, evidenced by an interaction between Block and CS type [*F*_(12, 432)_ = 3.88, *p* = 0.000, η^2^_p_ = 0.097]. A significant interaction between Sex and CS type showed that SCR responding to the different CS types was altered by Sex [*F*_(3, 108)_ = 3.30, *p* = 0.023, η^2^_p_ = 0.084], regardless of Drug. In addition, a significant three-way interaction between CS type, Sex, and Block showed that this relationship changed over the course of acquisition [*F*_(12, 432)_ = 2.40, *p* = 0.005, η^2^_p_ = 0.063]. The four-way interaction between CS type, Block, Sex, and Drug was not significant [*F*_(12, 432)_ = 1.41, *p* = *ns*.]. We did however observe a marginal significant three-way interaction between Block, Drug, and Sex [*F*_(4, 144)_ = 2.40, *p* = 0.053, η^2^_p_ = 0.062], indicating that collapsed over CS types, hydrocortisone differentially altered the course of general SCR responding for the two sexes. Finally, we observed a significant between-subjects effect of Drug [*F*_(1, 36)_ = 5.31, *p* = 0.027, η^2^_p_ = 0.129], indicating that cortisol overall heightened SCR values. There was no between-subjects effect of Sex (*F* < 0.64, *p* = *ns*.), nor did any of the other possible interactions reach significance (all *F*s < 1.82, *p* = *ns*.). The absence of a significant interaction with Drug, CS type, and/or Sex notwithstanding, we ran a generalization analysis on the average over the entire acquisition of all stimulus types. Data from the entire acquisition phase was taken because SCR responding severely habituated toward the end of conditioning. In line with the previous analysis, we found a main effect of CS type [*F*_(3, 111)_ = 48.95, *p* = 0.000, η^2^_p_ = 0.570] indicating a downward generalization gradient in startle responding as stimulus divergence increased from A(CS1)+. However, the generalization pattern from A(CS1)+ through B(CS2)- did not differ across Drug and Sex [*F*_(3, 111)_ = 1.69, *p* = *ns*.]. Thus, SCR data did not reveal altered contextualization patterns due to cortisol. Figure 4 displays SCR data for the four different groups.

#### Contextual fear

Similar to the FPS analysis we aimed at investigating whether cortisol, sex or a combination of the two did modulate SCR responding to the occasion setting contexts. Resembling the FPS data, this analysis solely revealed a main effect of Context Type [*F*_(1, 37)_ = 6.50, *p* = 0.015, η^2^_p_ = 0.149], indicating that SCR responding to the threat occasion setting context A was generally higher than to the safety occasion setting context B, regardless of Sex or Drug. Further, there was only a main effect of Block [*F*_(9, 333)_ = 6.65, *p* = 0.000, η^2^_p_ = 0.152], pointing out general habituation of SCR responding. None of the other interactions reached significance (all *F*s < 2, *p* = *ns*.), neither were there any between subjects-effects significant (all *F*s < 0.22, *p* = *ns*.).

#### Additional SCR analyses

Though we did not find altered generalization patterns by cortisol, previous studies have found alterations in differential-conditioning as measured by SCR due to cortisol administration in men and women (Stark et al., 2006; Wolf et al., 2009; Tabbert et al., 2010). The inclusion of all different CS types in the previous analysis may have reduced power to find an effect of Drug on simple differential conditioning. To investigate the variable effects of hydrocortisone on simple differential conditioning for the two sexes, we calculated the difference score between A(CS1)+ (the actual CS+) and B(CS2)− (safe CS in safe context) for each block of the entire acquisition. These blocks were entered in a mixed ANOVA with CS type and Block as the within-subjects factors, and Sex and Drug as the between-subjects factors. Like before, this analysis revealed a main effect of Block [*F*_(4, 144)_ = 7.48, *p* = 0.000, η^2^_p_ = 0.172], underlining that differential conditioning decreased toward the end of acquisition. Importantly, this analysis revealed a marginally significant interaction effect between Sex and Drug, pointing out that hydrocortisone indeed differently influenced conditioning for the two sexes [*F*_(1, 36)_ = 3.11, *p* = 0.086, η^2^_p_ = 0.080]. The between-subjects effect of Sex was significant as well [*F*_(1, 36)_ = 6.84, *p* = 0.013, η^2^_p_ = 0.160], showing that differential conditioning over the entire acquisition was higher for the women. Follow-up ANOVA's showed that collapsed over all blocks, hydrocortisone slightly impaired differential conditioning in men [*F*_(1, 36)_ = 2.72, *p* = 0.054, η^2^_p_ = 0.070], but seemed to have no effect on the women [*F*_(1, 36)_ = 0.712, *p* = *ns*.].

## Discussion

Here, we investigated the effect of cortisol on the contextualization of fear expression in men and women. A new paradigm was employed that incorporated context as a modulator of fear expression. The results indicated that people acquire differential contextual modulation of the expression of fear, as measured by FPS and SCR. In addition, while acute hydrocortisone treatment did not affect the US-expectancies, it impaired the contextualization of fear expression leading to increased fear generalization on FPS data in women. The opposite pattern was found in the male participants. Finally, cortisol impaired differential-conditioning in the male participants as measured by SCR.

One hypothesis of the etiology of posttraumatic disorder poses that extinction of conditioned fear is deficient in PTSD (Orr et al., 2003; Milad et al., 2006b; Rauch et al., 2006). Neuroimaging studies have shown that impairment in extinction recall is associated with reduced hippocampal activity (Kalisch et al., 2006; Milad et al., 2007), and as such, many neurocircuitry models of the etiology of anxiety disorders have -among other regions- incorporated the hippocampus. These views all emphasize that the hippocampus underlies the deficits in appreciation of safe contexts, during or even after *extinction* learning. However, other views emphasize dysregulation in the *acquisition* of contextualization of fearful memories as an important vulnerability factor for developing PTSD (Liberzon and Sripada, 2008; Cohen et al., 2009; Acheson et al., 2011). A strong asymmetry in the context-specificity of fear acquisition and extinction exists: as a rule, extinction is more context specific than acquisition (Bouton, 2002). This appears to be the case because extinction per definition is the second thing learned about the CS (Nelson, 2002). It is as if the learning and memory system encodes the second thing learned about a stimulus as a conditional, context-specific exception to the rule (Bouton, 2002). The present paradigm may overcome this problem as excitatory learning and inhibitory learning take place in parallel. Thus, by using a hippocampus-dependent task and incorporating contexts as modulators of threat and safety, the present paradigm adds important attributes to current test models employed in the search for etiological mechanisms of anxiety disorders.

Though the current paradigm clearly bears resemblance to prototypical occasion setting paradigms, it differs on some important features. In a Pavlovian sequential feature positive discrimination task, a conditioned stimulus A (the “target” stimulus) is followed by presentation of an US only if the target A is preceded by another stimulus X (the “feature” stimulus), represented as X→A+/A−. Here, feature X becomes a facilitator, or positive occasion setter, that controls the behavioral expression of the association between the target A and the US. Because we aimed at enhancing the face validity of our paradigm in terms of contextual modulation of discrete CSs, our positive occasion setter constituted an actual context, in the sense that is was present both before and after CS presentation. However, as the associative strength with the US of an occasion setter increases along with closer temporal contiguity to the CS (Swartzentruber, 1995), and also to prevent the perception of the stimulus combination as a simple compound, we presented another context (B) with the threat CS1. Over and above this control stimulus combination, we added a control combination (the safe CS2 in the safe context B) that had not even remotely been associated with the US, so that we could both 1) assess a generalization curve across stimulus types and 2) distinguish contextual discriminatory learning from simple differential conditioning. In animal studies, contexts that modulated the relationship between a discrete cue and the US did not acquire any associative property with the US (Bouton and Swartzentruber, 1986), suggesting that contexts are relatively-independent of a direct association with the US (Swartzentruber, 1995). This is the first human study testing the associative value of an occasion setting context employing physiological data. In contrast with some animal studies, present results showed that the threat context elicited more fear than the safe context. Therefore our results are in line with the idea that contexts acquire a direct association with the US (Rescorla and Wagner, 1972), despite being completely-independent of the US (Baeyens et al., 2001). In that sense, the enhanced fear to the context may reflect context conditioning, which is defined as the display of anxiety in a context in which shocks have been previously administered. Animal research suggests that context conditioning, as opposed to explicit cued fear-conditioning, captures features of a more sustained anxiety response (Walker et al., 2003). This has been shown in human research (e.g., Baas et al., 2004) as well. However, human studies modeling anxiety have typically done so by presenting participants with unpredictable shocks in certain contexts (Baas et al., 2004). Here we show that occasion setters as defined by a context in a predictable fear learning setting do acquire associative properties, even though they have never been directly associated with any US.

Animal research has begun to explore how stress or corticosteroids may alter the contextualization of fear memories. One study showed that exposure to extreme stress impaired contextual modulation of fear responses (Cohen et al., 2009). In an elegant study, it has been shown in mice that the induction of glucocorticoids into the hippocampus after fear-conditioning decreased the ability to restrict fear to the appropriate context (Kaouane et al., 2012). In line with these studies, here we hypothesized that cortisol may alter contextualization of fear expression. The present results indicate that cortisol reduced the contextualization of fear in women resulting in enhanced fear generalization both to threatening cues in different contexts, but also to safe cues within the threatening context. An opposite pattern was found for men: cortisol enhanced differential contextual processing. Importantly, FPS data showed that cortisol did not alter overall differential conditioning, thus it can be concluded that the findings are specifically attributable to the contextualization and generalization of fear. In general, these findings are in line with several animal studies (Bangasser and Shors, 2004; Weiss et al., 2005), showing facilitating effects of corticosteroids in males but impairing effects in females on hippocampal-dependent tasks. These divergent effects of cortisol in women may provide clues concerning sex specific vulnerabilities for stress related illnesses such as depression or anxiety disorders (Kessler et al., 1993; Elzinga and Bremner, 2002).

Despite vast interest in sex as an important modulator in development of stress-related pathology, the interaction between sex, cortisol, and emotional learning has hardly been studied in humans so far (Merz et al., 2010). Those rare human studies that have actually investigated these factors focused exclusively on simple associative learning (Zorawski et al., 2005, 2006; Stark et al., 2006; Merz et al., 2010, in press; Tabbert et al., 2010), and divergent effects have been found. Apart from sex differences in experimental effects of cortisol, the present study revealed considerable sex differences within the placebo group as well. Most notably, baseline FPS responses were higher for women than for man. The majority of fear-conditioning studies employing this measure includes both men and women as participants, but did not test for differences between the sexes (Baas et al., 2004). Other studies did test sex differences, but did not reveal any effects (Grillon, 2002). Finally, one study seems to fit the current finding of elevated startle responses in women (Grillon et al., 2004). Corroborating this finding, female rats showed elevated (unconditioned) startle responses as compared with males (De Jongh et al., 2005). Literature on sex differences in SCR is even less consistent, with studies showing enhanced differential conditioning in males (Milad et al., 2006a), showing no differences between the sexes (Zorawski et al., 2005), or revealing even enhanced responding in females (Guimarães et al., 1991). Here, we did not find any sex differences, but this may have been due to small sample size. Furthermore, it is becoming increasingly clear that hormonal status (e.g., estradiol, progesterone) of females can exert profound effects on the way fear is being expressed and recalled: high estradiol levels have been shown to be associated with enhanced extinction recall (Milad et al., 2010), while the suppression of endogenous sex hormones by OC can alter the neural activity during extinction learning (Merz et al., 2011). Arguably, interactions between sex and stress hormone levels can have important consequences for fear learning and expression. Taken together, future studies should carefully formulate their research questions taking sex differences into account, and refine their experimental designs accordingly. Ultimately, such an approach can have important implications for understanding the etiology and treatment of anxiety disorders (Lebron-Milad and Milad, 2012).

One other important, but probably undervalued, modulatory factor in the effects of stress on emotional learning is awareness of the experimental contingencies. Awareness is known to be an important modulator of fear expression (Grillon, 2002; Jovanovic et al., 2006), and is perhaps even a necessary condition to acquire conditioned responses in hippocampus-dependent tasks (Weike et al., 2007). Cortisol administration has been shown to increase the likelihood of the usage of a simple stimulus-response learning strategy at the cost of more complicated hippocampally-mediated spatial strategy (Schwabe et al., 2009b). Here, we explicitly did not want to complicate our conclusions by possible alterations in awareness due to corticosteroids. The only human study that employed an occasion setting paradigm along with a social stress manipulation did not find significantly more unaware participants in the experimental group (Wolf et al., 2012). Notably however, the authors did not incorporate awareness as an additional factor in their analyzes, and could therefore have missed important effects. Thus, the possibility of enhanced fear generalization due to the effects of stress hormones on awareness could be of potential interest for future studies.

In humans, little is known about the effects of cortisol on conditioning tasks using FPS. One study aimed at dissociating cortisol effects on fear from anxiety (Grillon et al., 2011). It was found that cortisol specifically potentiated anxiety, presumably by activating CRH receptors in the bed nucleus of the stria terminalis (BNST). However, an alternative interpretation for those findings could be that cortisol influenced brain areas involved in the processing of contextual cues, as opposed to explicit threat cues. Animal studies have indeed found selective effects of corticosteroids on context conditioning as opposed to cued conditioning (Pugh et al., 1997). Here, we did not find altered responses by cortisol on the contexts themselves. This may have been caused by relative small n size. Another explanation could be that the current dose of hydrocortisone employed was 20 mg as opposed to 60 mg that Grillon and colleagues employed (Grillon et al., 2011). Also, we did not observe altered baseline FPS startle responding (i.e., as measured on trials in between context presentation), in contrast with one study who found heightened FPS due to cortisol (Buchanan et al., 2001). We did however reveal contextual modulation of fear expression to discrete CSs, presumably through altered hippocampal processing. On a broader level, this finding is in line with research showing that stress hormones such as cortisol can switch the brain to a negative response bias of ambiguous cues (Enkel et al., 2010), or reduce amygdalar reactivity to positive faces while enhancing activity to negative faces (Kukolja et al., 2008) or even lead to simple cued driven behavioral strategies (Schwabe et al., 2007, 2008, 2009b). While all of these studies employed rather disparate paradigms as well as a divergence of dependent variables, they all underscore the idea that stress may become maladaptive and precipitate severe affective spectrum disorders including anxiety and/or major depression. To follow-up on the present study, an important next step would be to test the *retention* of fear contextualization after corticosteroid exposure, in line with animal studies showing impaired recall of contextual specificity of fear due to stress (Cohen et al., 2009) or corticosteroids (Kaouane et al., 2012). In humans, social stress has been shown to impair the contextual dependency of declarative memories (Schwabe et al., 2009a), but this study so far has been the only one of its kind. In conclusion, much more insight may be gained by showing that stress hormones not only alter acute contextual modulation of fear, but also long-term fear contextualization.

Even though the present FPS data converge on animal data, they seem to contrast with other human fear-conditioning studies where facilitating effects of cortisol on differential conditioning in women have been found (Stark et al., 2006; Merz et al., 2010; Tabbert et al., 2010), but impairing effects in men (Stark et al., 2006). Apart from the fact that qualitatively different paradigms were used, these seemingly contradictory observations can be explained by the fact that none of those studies used FPS as dependent measure. In the present study both SCR and FPS were employed, allowing for direct comparison of these variables. Focusing just on SCR, cortisol did impair differential conditioning in men, in line with other studies showing similar effects (Stark et al., 2006; Merz et al., 2010; Tabbert et al., 2010). This once again corroborates the idea that SCR and FPS variables reflect rather distinctive aspects of conditioned responses, and can sometimes even display diametrically opposite effects after certain experimental manipulations (Hamm and Weike, 2005; Soeter and Kindt, 2010, 2011, 2012; Sevenster et al., 2012). Importantly, we believe that the inclusion of the startle measure is a valuable addition to translational basic research aimed at bridging the gap between experimental findings and clinical understanding. The FPS is considered to be a reliable and specific index of fear, while SCR and US-expectancy ratings appear to be less so (Hamm and Weike, 2005). Indeed, many manipulations that target fear or anxiety can impact FPS as well. For instance, pharmacological agents targeting anxiety such as benzodiazepam reduce both startle responses and measures of subjective anxiety during periods of threat (i.e., shock) (Grillon et al., 2003, 2006). The current finding that cortisol amplifies fear generalization as indexed by fear potentiated starte data may indicate that cortisol (or stress) at a certain point may become maladaptive and in the end perhapse precipitate clincical anxiety.

Some important limitations of the present study should be mentioned. First of all, sample size was relatively small. With a larger sample size, perhaps we could have shown enhanced differential fear-conditioning on SCR data in women, similar to other studies (Jackson et al., 2006; Tabbert et al., 2010). On the other hand, effect sizes were rather strong in the present study. Furthermore, though this was not an instructed fear paradigm, instructions to the participants were rather explicit. Such instructions along with the continuous online US-expectancy ratings may have directed attention toward the CS–US relationships (Lovibond and Shanks, 2002). As SCR is highly sensitive to controlled, attentional processes (Filion et al., 1991), this methodological difference may have influenced SCR data and hamper comparison with SCR data of other studies employing less explicit instructions. Furthermore, a dose of 20 mg hydrocortisone was used. Several studies have shown that experimental effects can alter (or even flip over to the opposite side) along with variations in dose (Buchanan et al., 2001; Abercrombie et al., 2003), and dose-response studies are considered to be an auspicious way to investigate the specific effects of cortisol (Lupien et al., 1999). Another point of consideration is that numerous studies have reported sex differences in perceiving, interpreting, and reacting to stimuli that convey threat (Cahill et al., 2001; Stroud et al., 2002). Therefore, it is conceivable that the two sexes processed the male angry CSs differently. However, the advantage of such stimuli over for example geometric figures is that these are less susceptible to cognitive control, and can be easier conditioned (Mineka and Ohman, 2002). Furthermore, hormonal status in women can modulate fear learning (Milad et al., 2006a), expression (Merz et al., in press) and retention (Milad et al., 2010), while OC can alter neural activity during learning (Merz et al., 2011). Since all women in the present study were using OC, caution must be taken when generalizing findings from the present study to free cycling women. Finally, we were interested in the acute effects of cortisol on fear expression. However, in order to construct a more valid experimental model for the etiology of anxiety disorders, retention of long-term fear expression should be tested as well.

## Conclusion

The capability to learn to predict upcoming aversive events by using cues in the environment is essential for the survival of an organism. Both animal and human studies illustrate that stress and concomitant stress hormones like cortisol are capable of altering simple associative fear learning (Rodrigues et al., 2009; Wolf et al., 2012), which is of importance for understanding the development of pathological fear observed in anxiety disorders (Korte, 2001). Importantly, the ability to form representations of the environment can be impaired by stress, or concomitant stress hormones. Possibly, this may have deleterious consequences: lack of differential contextual control in the expression of fear due to corticosteroids could result in increased generalization of fear to discrete cues. In the end, this could drive the fear system toward pathological conditions. Here, we showed that cortisol enhanced fear generalization over contexts in women, while the opposite pattern emerged in men. The present study incorporated effects of cortisol and sex on the contextualization of fear expression. By doing so, the study adds to translational basic research that ultimately may result in an enhanced understanding of the origin of anxiety disorders.

### Conflict of interest statement

The authors declare that the research was conducted in the absence of any commercial or financial relationships that could be construed as a potential conflict of interest.

## References

[B1] Abercrombie H. C. Kalin N. H. Thurow M. E. Rosenkranz M. A. Davidson R. J. (2003). Cortisol variation in humans affects memory for emotionally laden and neutral information. Behav. Neurosci. 117, 505–516 12802879 10.1037/0735-7044.117.3.505

[B2] Acheson D. T. Gresack J. E. Risbrough V. B. (2011). Hippocampal dysfunction effects on context memory: possible etiology for post-traumatic stress disorder. Neuropharmacology 62, 674–685 10.1016/j.neuropharm.2011.04.029 21596050 PMC3175276

[B3] Baas J. M. Nugent M. Lissek S. Pine D. S. Grillon C. (2004). Fear conditioning in virtual reality contexts: a new tool for the study of anxiety. Biol. Psychiatry 55, 1056–1060 10.1016/j.biopsych.2004.02.024 15158423

[B4] Baeyens F. Vansteenwegen D. Hermans D. Vervliet B. Eelen P. (2001). Sequential and simultaneous feature positive discriminations: occasion setting and configural learning in human Pavlovian conditioning. J. Exp. Psychol. Anim. Behav. Process. 27, 279–295 11497326

[B5] Baeyens F. Vervliet B. Vansteenwegen D. Beckers T. Hermans D. Eelen P. (2004). Simultaneous and sequential feature negative discriminations: elemental learning and occasion setting in human Pavlovian conditioning. Learn. Motiv. 35, 136–166 11497326

[B6] Bangasser D. A. Shors T. J. (2004). Acute stress impairs trace eye blink conditioning in females without altering the unconditioned response. Neurobiol. Learn. Mem. 82, 57–60 10.1016/j.nlm.2004.03.001 15183171 PMC3363961

[B7] Barbas H. Saha S. Rempel-Clower N. Ghashghaei T. (2003). Serial pathways from primate prefrontal cortex to autonomic areas may influence emotional expression. BMC Neurosci. 4, 25 10.1186/1471-2202-4-25 14536022 PMC270042

[B8] Blumenthal T. D. Cuthbert B. N. Filion D. L. Hackley S. Lipp O. V. Van Boxtel A. (2005). Committee report: guidelines for human startle eyeblink electromyographic studies. Psychophysiology 42, 1–15 10.1111/j.1469-8986.2005.00271.x 15720576

[B9] Bouton M. E. (2002). Context, ambiguity, and unlearning: sources of relapse after behavioral extinction. Biol. Psychiatry 52, 976–986 12437938 10.1016/s0006-3223(02)01546-9

[B10] Bouton M. E. Swartzentruber D. (1986). Analysis of the associative and occasion-setting properties of contexts participating in a Pavlovian discrimination. J. Exp. Psychol. Anim. Behav. Process. 12, 333–350

[B11] Bradley M. M. Lang P. J. (1994). Measuring emotion: the self-assessment manikin and the semantic differential. J. Behav. Ther. Exp. Psychiatry 25, 49–59 7962581 10.1016/0005-7916(94)90063-9

[B12] Buchanan T. W. Brechtel A. Sollers J. J. Lovallo W. R. (2001). Exogenous cortisol exerts effects on the startle reflex independent of emotional modulation. Pharmacol. Biochem. Behav. 68, 203–210 10.1016/S0091-3057(00)00450-0 11267624

[B13] Cahill L. (2006). Why sex matters for neuroscience. Nat. Rev. Neurosci. 7, 477–484 10.1038/nrn1909 16688123

[B14] Cahill L. Haier R. J. White N. S. Fallon J. Kilpatrick L. Lawrence C. (2001). Sex-related difference in amygdala activity during emotionally influenced memory storage. Neurobiol. Learn. Mem. 75, 1–9 10.1006/nlme.2000.3999 11124043

[B15] Cohen H. Liberzon I. Richter-Levin G. (2009). Exposure to extreme stress impairs contextual odour discrimination in an animal model of PTSD. Int. J. Neuropsychopharmacol. 12, 291–303 10.1017/S146114570800919X 18700055

[B16] Cohen S. Kamarck T. Mermelstein R. (1983). A global measure of perceived stress. J. Health Soc. Behav. 24, 385–396 6668417

[B17] Cordero M. Venero C. Kruyt N. Sandi C. (2003). Prior exposure to a single stress session facilitates subsequent contextual fear conditioning in rats: evidence for a role of corticosterone. Horm. Behav. 44, 338–345 10.1016/S0018-506X(03)00160-0 14613728

[B18] Czock D. Keller F. Rasche F. M. Häussler U. (2005). Pharmacokinetics and pharmacodynamics of systemically administered glucocorticoids. Clin. Pharmacokinet. 44, 61–98 15634032 10.2165/00003088-200544010-00003

[B19] Dalla C. Shors T. J. (2009). Sex differences in learning processes of classical and operant conditioning. Physiol. Behav. 97, 229–238 10.1016/j.physbeh.2009.02.035 19272397 PMC2699937

[B20] De Houwer J. Crombez G. Baeyens F. (2005). Avoidance behavior can function as a negative occasion setter. J. Exp. Psychol. Anim. Behav. Process. 31, 101–106 10.1037/0097-7403.31.1.101 15656731

[B21] De Jongh R. Geyer M. A. Olivier B. Groenink L. (2005). The effects of sex and neonatal maternal separation on fear-potentiated and light-enhanced startle. Behav. Brain Res. 161, 190–196 10.1016/j.bbr.2005.02.004 15878207

[B22] De Vries J. (1998). The Perceived Stress Scale, Dutch Translation. Tilburg, Netherlands: Tilburg University

[B23] Duncko R. Cornwell B. Cui L. Merikangas K. R. Grillon C. (2007). Acute exposure to stress improves performance in trace eyeblink conditioning and spatial learning tasks in healthy men. Learn. Mem. 14, 329–335 10.1101/lm.483807 17522023 PMC1876756

[B24] Effting M. Kindt M. (2007). Contextual control of human fear associations in a renewal paradigm. Behav. Res. Ther. 45, 2002–2018 10.1016/j.brat.2007.02.011 17451643

[B25] Ehlers A. Clark D. M. (2000). A cognitive model of posttraumatic stress disorder. Behav. Res. Ther. 38, 319–345 10.1016/S0005-7967(99)00123-0 10761279

[B26] Elzinga B. M. Bremner J. D. (2002). Are the neural substrates of memory the final common pathway in posttraumatic stress disorder (PTSD)? J. Affect. Disord. 70, 1–17 12113915 10.1016/s0165-0327(01)00351-2 PMC5580811

[B27] Enkel T. Gholizadeh D. Von Bohlen Und Halbach O. Sanchis-Segura C. Hurlemann R. Spanagel R. (2010). Ambiguous-cue interpretation is biased under stress- and depression-like states in rats. Neuropsychopharmacology 35, 1008–1015 10.1038/npp.2009.204 20043002 PMC3055368

[B28] Filion D. L. Dawson M. E. Schell A. M. Hazlett E. A. (1991). The relationship between skin conductance orienting and the allocation of processing resources. Psychphysiology 28, 410–424 1745721 10.1111/j.1469-8986.1991.tb00725.x

[B29] Fridlund A. J. Cacioppo J. T. (1986). Guidelines for human electromyographic research. Psychophysiology 23, 567–589 3809364 10.1111/j.1469-8986.1986.tb00676.x

[B30] Grillon C. (2002). Associative learning deficits increase symptoms of anxiety in humans. Biol. Psychiatry 51, 851–858 12022957 10.1016/s0006-3223(01)01370-1

[B31] Grillon C. (2008). Models and mechanisms of anxiety: evidence from startle studies. Psychopharmacology 199, 421–437 10.1007/s00213-007-1019-1 18058089 PMC2711770

[B32] Grillon C. Baas J. P. Lissek S. Smith K. Milstein J. (2004). Anxious responses to predictable and unpredictable aversive events. Behav. Neurosci. 118, 916–924 10.1037/0735-7044.118.5.916 15506874

[B33] Grillon C. Baas J. M. P. Pine D. S. Lissek S. Lawley M. Ellis V. (2006). The benzodiazepine alprazolam dissociates contextual fear from cued fear in humans as assessed by fear-potentiated startle. Biol. Psychiatry 60, 760–766 10.1016/j.biopsych.2005.11.027 16631127

[B34] Grillon C. Cordova J. Levine L. R. Morgan C. A. (2003). Anxiolytic effects of a novel group II metabotropic glutamate receptor agonist (LY354740) in the fear-potentiated startle paradigm in humans. Psychopharmacology 168, 446–454 10.1007/s00213-003-1444-8 12709777

[B35] Grillon C. Heller R. Hirschhorn E. Kling M. A. Pine D. S. Schulkin J. (2011). Acute hydrocortisone treatment increases anxiety but not fear in healthy volunteers: a fear-potentiated startle study. Biol. Psychiatry 69, 1–7 10.1016/j.biopsych.2010.12.013 21277566 PMC3116445

[B36] Guimarães F. S. Hellewell J. Hensman R. Wang M. Deakin J. F. (1991). Characterization of a psychophysiological model of classical fear conditioning in healthy volunteers: influence of gender, instruction, personality and placebo. Psychopharmacology 104, 231–236 1876667 10.1007/BF02244184

[B37a] Hannan D. (1999). Coral Sea Dreaming. Mountain Lakes, NJ: DVD International

[B37] Hamm A. O. Weike A. I. (2005). The neuropsychology of fear learning and fear regulation. Int. J. Psychophysiol. 57, 5–14 10.1016/j.ijpsycho.2005.09.005 15935258

[B38] Hartley C. A. Phelps E. A. (2009). Changing fear: the neurocircuitry of emotion regulation. Neuropsychopharmacology 35, 136–146 10.1038/npp.2009.121 19710632 PMC3055445

[B39] Herman J. P. Ostrander M. M. Mueller N. K. Figueiredo H. (2005). Limbic system mechanisms of stress regulation: hypothalamo-pituitary-adrenocortical axis. Prog. Neuropsychopharmacol. Biol. Psychiatry 29, 1201–1213 10.1016/j.pnpbp.2005.08.006 16271821

[B40] Het S. Ramlow G. Wolf O. T. (2005). A meta-analytic review of the effects of acute cortisol administration on human memory. Psychoneuroendocrinology 30, 771–784 10.1016/j.psyneuen.2005.03.005 15919583

[B41] Jackson E. D. Payne J. D. Nadel L. Jacobs W. J. (2006). Stress differentially modulates fear conditioning in healthy men and women. Biol. Psychiatry 59, 516–522 10.1016/j.biopsych.2005.08.002 16213468

[B42] Joëls M. Baram T. (2009). The neuro-symphony of stress. Nat. Rev. Neurosci. 10, 459–466 10.1038/nrn2632 19339973 PMC2844123

[B43] Joëls M. Karst H. Derijk R. De Kloet E. R. (2008). The coming out of the brain mineralocorticoid receptor. Trends Neurosci. 31, 1–7 10.1016/j.tins.2007.10.005 18063498

[B44a] Jovanovic T. Keyes M. Fiallos A. Myers K. M. Davis M. Duncan E. J. (2005). Fear potentiation and fear inhibition in a human fear-potentiated startle paradigm. Biol. Psychiatry 57, 1559–1564 10.1016/j.biopsych.2005.02.025 15953493

[B44] Jovanovic T. Norrholm S. D. Keyes M. Fiallos A. Jovanovic S. Myers K. M. (2006). Contingency awareness and fear inhibition in a human fear-potentiated startle paradigm. Behav. Neurosci. 120, 995–1004 10.1037/0735-7044.120.5.995 17014251 PMC3740393

[B45] Kalisch R. Korenfeld E. Stephan K. Weiskopf N. Seymour B. Dolan R. (2006). Context-dependent human extinction memory is mediated by a ventromedial prefrontal and hippocampal network. J. Neurosci. 26, 9503 10.1523/JNEUROSCI.2021-06.2006 16971534 PMC2634865

[B46] Kaouane N. Porte Y. Vallée M. Brayda-Bruno L. Mons N. Calandreau L. (2012). Glucocorticoids can induce PTSD-like memory impairments in mice. Science 335, 1510–1513 10.1126/science.1207615 22362879

[B47] Kessler R. C. McGonagle K. A. Swartz M. Blazer D. G. Nelson C. B. (1993). Sex and depression in the National Comorbidity Survey. I: lifetime prevalence, chronicity and recurrence. J. Affect. Disord. 29, 85–96 10.1016/0165-0327(93)90026-G 8300981

[B48] Kindt M. Soeter M. Vervliet B. (2009). Beyond extinction: erasing human fear responses and preventing the return of fear. Nat. Neurosci. 12, 256–258 10.1038/nn.2271 19219038

[B49] Kohn P. M. Macdonald J. E. (1992). The survey of recent life experiences: a decontaminated hassles scale for adults. J. Behav. Med. 15, 221–236 1583682 10.1007/BF00848327

[B50] Korte S. (2001). Corticosteroids in relation to fear, anxiety and psychopathology. Neurosci. Biobehav. Rev. 25, 117–142 10.1016/S0149-7634(01)00002-1 11323078

[B51] Kuehl L. K. Lass-Hennemann J. Richter S. Blumenthal T. D. Oitzl M. Schachinger H. (2010). Accelerated trace eyeblink conditioning after cortisol IV-infusion. Neurobiol. Learn. Mem. 94, 547–553 10.1016/j.nlm.2010.09.007 20850556

[B52] Kukolja J. Schläpfer T. E. Keysers C. Klingmüller D. Maier W. Fink G. R. (2008). Modeling a negative response bias in the human amygdala by noradrenergic-glucocorticoid interactions. J. Neurosci. 28, 12868–12876 10.1523/JNEUROSCI.3592-08.2008 19036981 PMC6671813

[B53] Labar K. S. Cook C. A. Torpey D. C. Welsh-Bohmer K. A. (2004). Impact of healthy aging on awareness and fear conditioning. Behav. Neurosci. 118, 905–915 10.1037/0735-7044.118.5.905 15506873

[B54] Lebron-Milad K. Milad M. R. (2012). Sex differences, gonadal hormones and the fear extinction network: implications for anxiety disorders. Biol. Mood Anxiety Disord. 2, 3 10.1186/2045-5380-2-3 22738383 PMC3384233

[B55] Liberzon I. Sripada C. (2008). The functional neuroanatomy of PTSD: a critical review. Prog. Brain Res. 167, 151–169 10.1016/S0079-6123(07)67011-3 18037013

[B56] Lissek S. Biggs A. Rabin S. Cornwell B. Alvarez R. Pine D. (2008). Generalization of conditioned fear-potentiated startle in humans: experimental validation and clinical relevance. Behav. Res. Ther. 46, 678–687 10.1016/j.brat.2008.02.005 18394587 PMC2435484

[B57] Lissek S. Rabin S. Heller R. E. Lukenbaugh D. Geraci M. Pine D. S. (2010). Overgeneralization of conditioned fear as a pathogenic marker of panic disorder. Am. J. Psychiatry 167, 47–55 10.1176/appi.ajp.2009.09030410 19917595 PMC2806514

[B58] Lovibond P. F. Shanks D. R. (2002). The role of awareness in Pavlovian conditioning: empirical evidence and theoretical implications. J. Exp. Psychol. Anim. Behav. Process. 28, 3–26 11868231

[B59] Lupien S. J. Gillin C. J. Hauger R. L. (1999). Working memory is more sensitive than declarative memory to the acute effects of corticosteroids: a dose-response study in humans. Behav. Neurosci. 113, 420–430 10443770 10.1037//0735-7044.113.3.420

[B60] Lupien S. J. Wilkinson C. W. Brière S. Ménard C. Ng Ying Kin N. M. K. Nair N. P. V. (2002). The modulatory effects of corticosteroids on cognition: studies in young human populations. Psychoneuroendocrinology 27, 401–416 10.1016/S0306-4530(01)00061-0 11818174

[B61] Lykken D. T. (1972). Range correction applied to heart rate and to GSR data. Psychophysiology 9, 373–379 5034126 10.1111/j.1469-8986.1972.tb03222.x

[B62] Majella De Jong G. Timmerman I. G. H. Emmelkamp P. M. G. (1996). The survey of recent life experiences: a psychometric evaluation. J. Behav. Med. 19, 529–542 8970913 10.1007/BF01904902

[B63] Merz C. J. Stark R. Vaitl D. Tabbert K. Wolf O. T. (in press). Stress hormones are associated with the neuronal correlates of instructed fear conditioning. Biol. Psychol. 10.1016/j.biopsycho.2012.02.017 22406758

[B64] Merz C. J. Tabbert K. Schweckendiek J. Klucken T. Vaitl D. Stark R. (2010). Investigating the impact of sex and cortisol on implicit fear conditioning with fMRI. Psychoneuroendocrinology 35, 33–46 10.1016/j.psyneuen.2009.07.009 19683399

[B65] Merz C. J. Tabbert K. Schweckendiek J. Klucken T. Vaitl D. Stark R. (2011). Neuronal correlates of extinction learning are modulated by sex hormones. Soc. Cogn. Affect. Neurosci. [Epub ahead of print]. 10.1093/scan/nsr063 21990419 PMC3475362

[B66] Milad M. Orr S. Pitman R. Rauch S. (2005). Context modulation of memory for fear extinction in humans. Psychophysiology 42, 456–464 10.1111/j.1469-8986.2005.00302.x 16008774

[B67] Milad M. R. Goldstein J. M. Orr S. P. Wedig M. M. Klibanski A. Pitman R. K. (2006a). Fear conditioning and extinction: influence of sex and menstrual cycle in healthy humans. Behav. Neurosci. 120, 1196–1203 10.1037/0735-7044.120.5.1196 17201462

[B68] Milad M. R. Rauch S. L. Pitman R. K. Quirk G. J. (2006b). Fear extinction in rats: implications for human brain imaging and anxiety disorders. Biol. Psychol. 73, 61–71 10.1016/j.biopsycho.2006.01.008 16476517

[B69] Milad M. R. Wright C. I. Orr S. P. Pitman R. K. Quirk G. J. Rauch S. L. (2007). Recall of fear extinction in humans activates the ventromedial prefrontal cortex and hippocampus in concert. Biol. Psychiatry 62, 446–454 10.1016/j.biopsych.2006.10.011 17217927

[B70] Milad M. R. Zeidan M. A. Contero A. Pitman R. K. Klibanski A. Rauch S. L. (2010). The influence of gonadal hormones on conditioned fear extinction in healthy humans. Neuroscience 168, 652–658 10.1016/j.neuroscience.2010.04.030 20412837 PMC2881679

[B71] Mineka S. Oehlberg K. (2008). The relevance of recent developments in classical conditioning to understanding the etiology and maintenance of anxiety disorders. Acta Psychol. 127, 567–580 10.1016/j.actpsy.2007.11.007 18226795

[B72] Mineka S. Ohman A. (2002). Phobias and preparedness: the selective, automatic, and encapsulated nature of fear. Biol. Psychiatry 52, 927–937 12437934 10.1016/s0006-3223(02)01669-4

[B73] Nees F. Richter S. Lass-Hennemann J. Blumenthal T. D. Schächinger H. (2008). Inhibition of cortisol production by metyrapone enhances trace, but not delay, eyeblink conditioning. Psychopharmacology 199, 183–190 10.1007/s00213-008-1155-2 18478206

[B74] Nelson J. B. (2002). Context specificity of excitation and inhibition in ambiguous stimuli. Learn. Motiv. 33, 284–310

[B75] Neumann D. L. Kitlertsirivatana E. (2010). Exposure to a novel context after extinction causes a renewal of extinguished conditioned responses: implications for the treatment of fear. Behav. Res. Ther. 48, 565–570 10.1016/j.brat.2010.03.002 20356572

[B76] Orr S. P. Metzger L. J. Lasko N. B. Macklin M. L. Hu F. B. Shalev A. Y. (2003). Physiologic responses to sudden, loud tones in monozygotic twins discordant for combat exposure: association with posttraumatic stress disorder. Arch. Gen. Psychiatry 60, 283–288 10.1001/archpsyc.60.3.283 12622661

[B78] Pruessner J. C. Wolf O. T. Hellhammer D. H. Buske-Kirschbaum A. Von Auer K. Jobst S. (1997). Free cortisol levels after awakening: a reliable biological marker for the assessment of adrenocortical activity. Life Sci. 61, 2539–2549 10.1016/S0024-3205(97)01008-4 9416776

[B79] Pugh C. R. Tremblay D. Fleshner M. Rudy J. W. (1997). A selective role for corticosterone in contextual-fear conditioning. Behav. Neurosci. 111, 503–511 9189265

[B80] Rauch S. L. Shin L. M. Phelps E. A. (2006). Neurocircuitry models of posttraumatic stress disorder and extinction: human neuroimaging research–past, present, and future. Biol. Psychiatry 60, 376–382 10.1016/j.biopsych.2006.06.004 16919525

[B81] Rescorla R. A. Wagner A. R. (1972). A theory of pavlovian conditioning, variations in the effectiveness of reinforcement and nonreinforcement, in Classical Conditioning II: Current Theory and Research, eds Black A. H. Prokasy W. F. (New York, NY: Appleton-Century-Crofts), 64–99

[B82] Rodrigues S. M. Ledoux J. E. Sapolsky R. M. (2009). The influence of stress hormones on fear circuitry. Annu. Rev. Neurosci. 32, 289–313 10.1146/annurev.neuro.051508.135620 19400714

[B83] Schiller D. Levy I. Niv Y. Ledoux J. E. Phelps E. A. (2008). From fear to safety and back: reversal of fear in the human brain. J. Neurosci. 28, 11517–11525 10.1523/JNEUROSCI.2265-08.2008 18987188 PMC3844784

[B84] Schmajuk N. A Buhusi C. V. (1997). Stimulus configuration, occasion setting, and the hippocampus. Behav. Neurosci. 111, 235–257 appendix 258. 9106665 10.1037//0735-7044.111.2.235

[B85] Schwabe L. Böhringer A. Wolf O. T. (2009a). Stress disrupts context-dependent memory. Learn. Mem. 16, 110–113 10.1101/lm.1257509 19181616

[B86] Schwabe L. Oitzl M. Richter S. Schächinger H. (2009b). Modulation of spatial and stimulus-response learning strategies by exogenous cortisol in healthy young women. Psychoneuroendocrinology 34, 358–366 10.1016/j.psyneuen.2008.09.018 18990499

[B87] Schwabe L. Dalm S. Schächinger H. Oitzl M. S. (2008). Chronic stress modulates the use of spatial and stimulus-response learning strategies in mice and man. Neurobiol. Learn. Mem. 90, 495–503 10.1016/j.nlm.2008.07.015 18707011

[B88] Schwabe L. Oitzl M. Philippsen C. Richter S. Bohringer A. Wippich W. (2007). Stress modulates the use of spatial versus stimulus-response learning strategies in humans. Learn. Mem. 14, 109 10.1101/lm.435807 17272656 PMC1838541

[B89] Sevenster D. Beckers T. Kindt M. (2012). Retrieval *per se* is not sufficient to trigger reconsolidation of human fear memory. Neurobiol. Learn. Mem. 97, 338–345 10.1016/j.nlm.2012.01.009 22406658

[B90] Shin L. Liberzon I. (2009). The neurocircuitry of fear, stress, and anxiety disorders. Neuropsychopharmacology 35, 169–191 10.1038/npp.2009.83 19625997 PMC3055419

[B91] Soeter M. Kindt M. (2010). Dissociating response systems: erasing fear from memory. Neurobiol. Learn. Mem. 94, 30–41 10.1016/j.nlm.2010.03.004 20381628

[B92] Soeter M. Kindt M. (2011). Stimulation of the noradrenergic system during memory formation impairs extinction learning but not the disruption of reconsolidation. Neuropsychopharmacology 18, 375–366 10.1038/npp.2011.307 22169947 PMC3306881

[B93] Soeter M. Kindt M. (2012). Erasing fear for an imagined threat event. Psychoneuroendocrinology 37, 1769–1779 10.1016/j.psyneuen.2012.03.011 22503387

[B94] Spielberger C. D. Gorsuch R. L. Lusthene R. E. (1970). Manual for the State-Trait Anxiety. Inventory. Palo Alto, CA: Consulting Psychologist Press

[B95] Stark R. Wolf O. T. Tabbert K. Kagerer S. Zimmermann M. Kirsch P. (2006). Influence of the stress hormone cortisol on fear conditioning in humans: evidence for sex differences in the response of the prefrontal cortex. Neuroimage 32, 1290–1298 10.1016/j.neuroimage.2006.05.046 16839780

[B96] Stroud L. R. Salovey P. Epel E. S. (2002). Sex differences in stress responses: social rejection versus achievement stress. Biol. Psychiatry 52, 318–327 12208639 10.1016/s0006-3223(02)01333-1

[B97] Swartzentruber D. (1995). Modulatory mechanisms in pavlovian conditioning. Anim. Learn. Behav. 23, 123–143

[B98] Tabbert K. Merz C. J. Klucken T. Schweckendiek J. Vaitl D. Wolf O. T. (2010). Cortisol enhances neural differentiation during fear acquisition and extinction in contingency aware young women. Neurobiol. Learn. Mem. 94, 392–401 10.1016/j.nlm.2010.08.006 20800102

[B99] Tottenham N. Tanaka J. W. Leon A. C. McCarry T. Nurse M. Hare T. A. (2009). The NimStim set of facial expressions: judgments from untrained research participants. Psychiatry Res. 168, 242–249 10.1016/j.psychres.2008.05.006 19564050 PMC3474329

[B100] Trentani A. Kuipers S. D. Te Meerman G. J. Beekman J. Ter Horst G. J. Den Boer J. A. (2003). Immunohistochemical changes induced by repeated footshock stress: revelations of gender-based differences. Neurobiol. Dis. 14, 602–618 10.1016/j.nbd.2003.08.006 14678775

[B101] Van Der Ploeg H. Defares P. Spielberger C. D. (1980). Handleiding bij de Zelf-Beoordelings Vragenlijst ZBV: een nederlandstalige bewerking van de Spielberger State-Trait Anxiety Inventory STAI-DY. Lisse: Swets and Zeitlinger

[B102] Vansteenwegen D. Hermans D. Vervliet B. Francken G. Beckers T. Baeyens F. (2005). Return of fear in a human differential conditioning paradigm caused by a return to the original acquistion context. Behav. Res. Ther. 43, 323–336 10.1016/j.brat.2004.01.001 15680929

[B103] Vythilingam M. Lawley M. Collin C. Bonne O. Agarwal R. Hadd K. (2006). Hydrocortisone impairs hippocampal-dependent trace eyeblink conditioning in post-traumatic stress disorder. Neuropsychopharmacology 31, 182–188 10.1038/sj.npp.1300843 16123770

[B104] Walker D. L. Toufexis D. J. Davis M. (2003). Role of the bed nucleus of the stria terminalis versus the amygdala in fear, stress, and anxiety. Eur. J. Pharmacol. 463, 199–216 10.1016/S0014-2999(03)01282-2 12600711

[B105] Watson D. Clark L. A. Tellegen A. (1988). Development and validation of brief measures of positive and negative affect: the PANAS scales. J. Pers. Soc. Psychol. 54, 1063–1070 3397865 10.1037//0022-3514.54.6.1063

[B106] Weike A. Schupp H. Hamm A. (2007). Fear acquisition requires awareness in trace but not delay conditioning. Psychophysiology 44, 170–180 10.1111/j.1469-8986.2006.00469.x 17241153

[B107] Weiss C. Sametsky E. Sasse A. Spiess J. Disterhoft J. F. (2005). Acute stress facilitates trace eyeblink conditioning in C57BL/6 male mice and increases the excitability of their CA1 pyramidal neurons. Learn. Mem. 12, 138–143 10.1101/lm.89005 15805311 PMC1074331

[B108] Wolf O. (2008). The influence of stress hormones on emotional memory: relevance for psychopathology. Acta Psychol. 127, 513–531 10.1016/j.actpsy.2007.08.002 17900515

[B109] Wolf O. T. Bauser D. S. Daum I. (2012). Eyeblink conditional discrimination learning in healthy young men is impaired after stress exposure. Psychophysiology 49, 164–171 10.1111/j.1469-8986.2011.01294.x 22091926

[B109a] Wolf O. T. Minnebusch D. Daum I. (2009). Stress impairs acquisition of delay eyeblink conditioning in men and women. Neurobiol. Learn. Mem. 91, 431–436 10.1016/j.nlm.2008.11.002 19049887

[B110] Zorawski M. Blanding N. Q. Kuhn C. M. Labar K. S. (2006). Effects of stress and sex on acquisition and consolidation of human fear conditioning. Learn. Mem. 13, 441–450 10.1101/lm.189106 16847304 PMC1538921

[B111] Zorawski M. Cook C. A. Kuhn C. M. Labar K. S. (2005). Sex, stress, and fear: individual differences in conditioned learning. Cogn. Affect. Behav. Neurosci. 5, 191–201 16180625 10.3758/cabn.5.2.191

